# Simultaneous detection of five flavoring agents in chewing gum by ultrasound-microwave synergistic extraction coupled with gas chromatography

**DOI:** 10.1038/s41598-019-48522-5

**Published:** 2019-08-19

**Authors:** Junde Li, Xiaojuan Liu, Xin Liang, Manman Zhang, Lei Han, Jiying Song

**Affiliations:** College of Chemistry and Pharmaceutical Sciences, Qingdao Agricultural University, 700 Changcheng Road, Qingdao, Shandong 266109 China

**Keywords:** Analytical chemistry, Chemical safety

## Abstract

So far, the identification and determination of flavor additives in food has gained extensive attention in the area of food safety. However, it remains a big challenge for simultaneous detection of diverse flavor agents. In this work, a novel gas chromatography method coupled with ultrasound-microwave synergistic extraction was developed for simultaneous detection of five flavor compounds, including butyl butyrate, menthol, methyl salicylate, eugenol and vanilline. In this strategy, ultrasound-microwave synergistic extraction was used to extract the five flavoring agents from chewing gum. The effects of extractants, solid-liquid ratio, extraction time and microwave power on extraction yield were researched by using orthogonal test. After the optimization of programme temperature and splitless injection, the five flavoring agents were well separated and simultaneously detected with wide linear ranges, low limits of detection, high accuracy and good repeatability. Therefore, this proposed method would hold great promise for assay application on the food safety.

## Introduction

Spices have been widely used as flavoring agents in food for improving the sensory quality and the flavor of foods. As a kind of food adjunct, the volatile spices compounds including butyl butyrate, menthol, vanilline, methyl salicylate and eugenol, are common flavoring additives, which are commonly used in toothpaste spices, tobacco spices and oral cleansers, due to their strong aroma and flavor. However, the intake of excessive flavoring agents is potentially hazard to human health^1^. Therefore, it is of great significant to accurately and reliably determine the flavoring agents in food for insuring safety.

So far, various analytical techniques have been reported to identify trace amounts of volatile compounds. Many conventional methods are based on chromatographic techniques, such as gas chromatography (GC), GC coupled with mass spectrometry (GC-MS), and high performance liquid chromatography (HPLC)^2–5^. Among them, GC and GC-MS have attracted growing interest in the qualitative identification and quantitative measurement of the volatile compounds. For example, some research groups reported that flavoring additives in food could also be detected by GC and GC-MS, such as menthol in food^6^, eugenol in fish and shrimp muscle tissue^7–10^ as well as vanillin in fruit juice and milk powder^1,11^. Additionally, GC and GC-MS have been extensively applied for other assay fields, such as determination of eugenol in traditional Chinese medicine^12^, menthol and methyl salicylate in human plasma^13^, vanillin and ethyl vanillin in vanilla extract products^14^, methyl salicylate in ulmus pumila leaves^15^, and menthol in traditional Chinese medicinal herbs^16^. Nevertherless, previously reported methods mainly focused on the detection of one flavor agent in food, and the challenging of the parallel detection of several flavor agents was rarely addressed. Thus, it is of great importance to develop a highly sensitive assay for multiple flavor agents in food.

It is essential to extract the trace flavor agents for enriching the analytes and removing the food interferences, since the content of flavoring agents in food is low. Hence, many extraction techniques were widely developed for the pre-concentration of food samples due to their great efficiency and reliability^8,17–19^, such as ultrasound extraction and microwave extraction. The ultrasound extraction can accelerate mass transfer from sample to extractive solvent^20,21^. On the other hand, microwave extraction can also prompte samples to dissolve into solvent. In recent years, ultrasound-microwave synergistic extraction has been developed as a novel technique for extraction and separation, due to the outstanding performance of ultrasound-microwave synergistic extraction, such as short extraction time, high extraction efficiency and low sample consumption^22,23^. Such unique advantages make it extensively exploit on medicine, chemistry and food.

In this contribution, we firstly developed a novel rapid and simple method based on combination of GC and ultrasound-microwave synergistic extraction technique, which was capable of extracting and detecting the diverse flavoring agents of food samples. In this work, we firstly used chewing gums as a proof-of-concept model to evaluate the procedure and conditions of extracting flavor. And then, five flavor agents were successfully extracted from the samples and simultaneously tested by this method. This proposed method would hold great promise for assay application on the food assay.

## Results and Discussion

### Extraction and enrichment of the flavor agents

Since the content of flavor agents added in chewing gums is low, the extraction and enrichment of these trace flavor agents are the crucial steps before the selective and sensitive detection. Here, as an effective technique to extract targets from food samples, ultrasound-microwave synergistic extraction, was used to extract and enrich flavor agents from chewing gum. In order to obtain the best performance of extraction and enrichment, several extraction conditions were optimized by the rationally designed orthogonal test. The extraction temperature was set at 60 °C, which was about 20 °C lower than the boiling point of each solvent in order to prevent violent boiling.

Orthogonal test is a design method of using the orthogonal table to arrange and analyze the multifactor test, which is from the whole level combination of test factors^24^. This test is carried out by selecting some representative horizontal combination and then the results of this part were analyzed to understand the situation of comprehensive test and select the optimal level combination. Orthogonal test is an efficient, fast, and economical experimental design method which basic characteristic is to use partial test instead of comprehensive test^24^. In this study, L_9_ orthogonal array (3)^4^ with four factors was designed and each factor had three levels. The four factors, including solvent, solid–liquid ratio, microwave power, and extraction time with their levels were listed in Table 1.

**Table 1:** The four factors with three levels for orthogonal test.

Levels	Factors			
	A (Solvent)	B (Solid-liquid ratio g:mL)	C (Microwave power/W)	D (Extraction time/min)
1	Ethanol	1:10	300	10
2	Ether	1:15	400	15
3	Petroleum ether	1:20	500	30

Nine sets of orthogonal test schemes were established in Table 2, where the peak area of methyl salicylate was used as the dependent variable. In chromatographic analysis, the peak area of the target was proportional to its concentration, and increased with the increase of its concentration. In Table 2, K_1_, K_2_, and K_3_ represented the sum of test results corresponding to different levels (1, 2 and 3) of Table 1. The optimal level of each factor was confirmed according K_i_ values. The higher the value of K_i_ was, the better this level was. The range R was the difference value of maximum of K_i_ and minimum of K_i_. The column with the largest R value in Table 2 corresponded to the uppermost factor. The results showed that the order of the importance of four factors was solvent, microwave power, extraction time, and solid-liquid ratio. In the orthogonal test analysis, levels with higher values favored higher extraction effect of the targets. Thus, the optimum extraction conditions for the targets were identified as the follows: ethanol as solvent, microwave power 400 W, extraction time 30 min, and solid–liquid ratio as 1:20 (g:mL). Then, these conditions were used to prepare the flavoring agent extracts in chewing gum and other food. Subsquently, the prepared flavoring agent extracts were identify by using the presented GC method.

**Table 2:** Results and analysis of L_9_(3)^4^ orthogonal test.

No.	Factors and their levels				Area
	A	B	C	D	
1	1	1	1	1	111616.5
2	1	2	2	2	359532.5
3	1	3	3	3	388751.5
4	2	1	2	3	370146.5
5	2	2	3	1	61953.5
6	2	3	1	2	52698.0
7	3	1	3	2	42163.0
8	3	2	1	3	31697.0
9	3	3	2	1	93872.0
K_1_	859900.5	523926.0	196011.5	267442.0	
K_2_	484798.0	453183.0	823551.0	454393.5	
K_3_	129782.0	535321.5	492868.0	790595.0	
R	730118.5	82138.5	627539.5	523153.0	
Primary and secondary order	A > C > D > B				
Excellent program	A_1_C_2_D_3_B_3_				

### Chromatogram of the Standards

**Figure 1:**
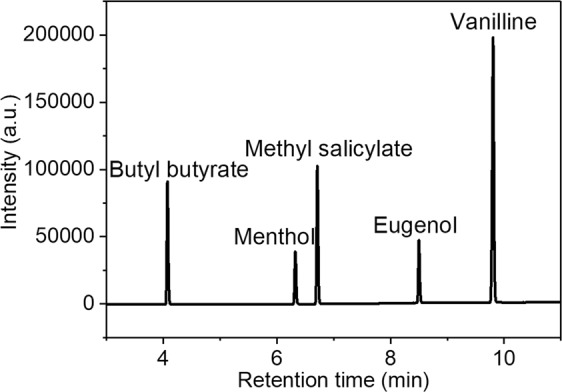
Gas chromatogram of mixed standard solutions.

To demonstrate the feasibility of identify volatile substances by using the present GC method, mixed standard solution was determined under the gas chromatograph conditions displayed in Table 3. The obtained gas chromatogram of mixed standard solutions was shown in Fig. 1. Obviously, sharp peaks corresponding to the five targets were obtained, indicating that the flavoring agents were adequately separated within 10 min. Elution sequence of five flavoring agents from the column were orderly butyl butyrate, menthol, methyl salicylate, eugenol and vanilline, respectively.

**Table 3:** Gas chromatograph conditions.

Parameters of GC condition	Setting			
Column	Rtx-1701, 30 m × 0.25 mm × 0.25 μm			
Inject port temperature	310 °C			
Detector	Flame ionization detector (FID)			
Detector temperature	310 °C			
Oven temperature programme		Rate (°C/min)	Temperature (°C)	Hold time (min)
	Initial	−	80	2
	1st	20	90	0
	2nd	40	130	0
	3rd	5	140	0
	4th	20	200	3
Run time	11.5 min			
Carrier gas	Nitrogen			
Carrier gas flow rate	30 mL min^−1^			
Detector gas flow rate	Hydrogen 40 mL min^−1^, air 400 mL min^−1^			
Injection volume	0.5 μL			
Injection mode	splitless

### Selectivity evaluation of standard mixtures

**Figure 2:**
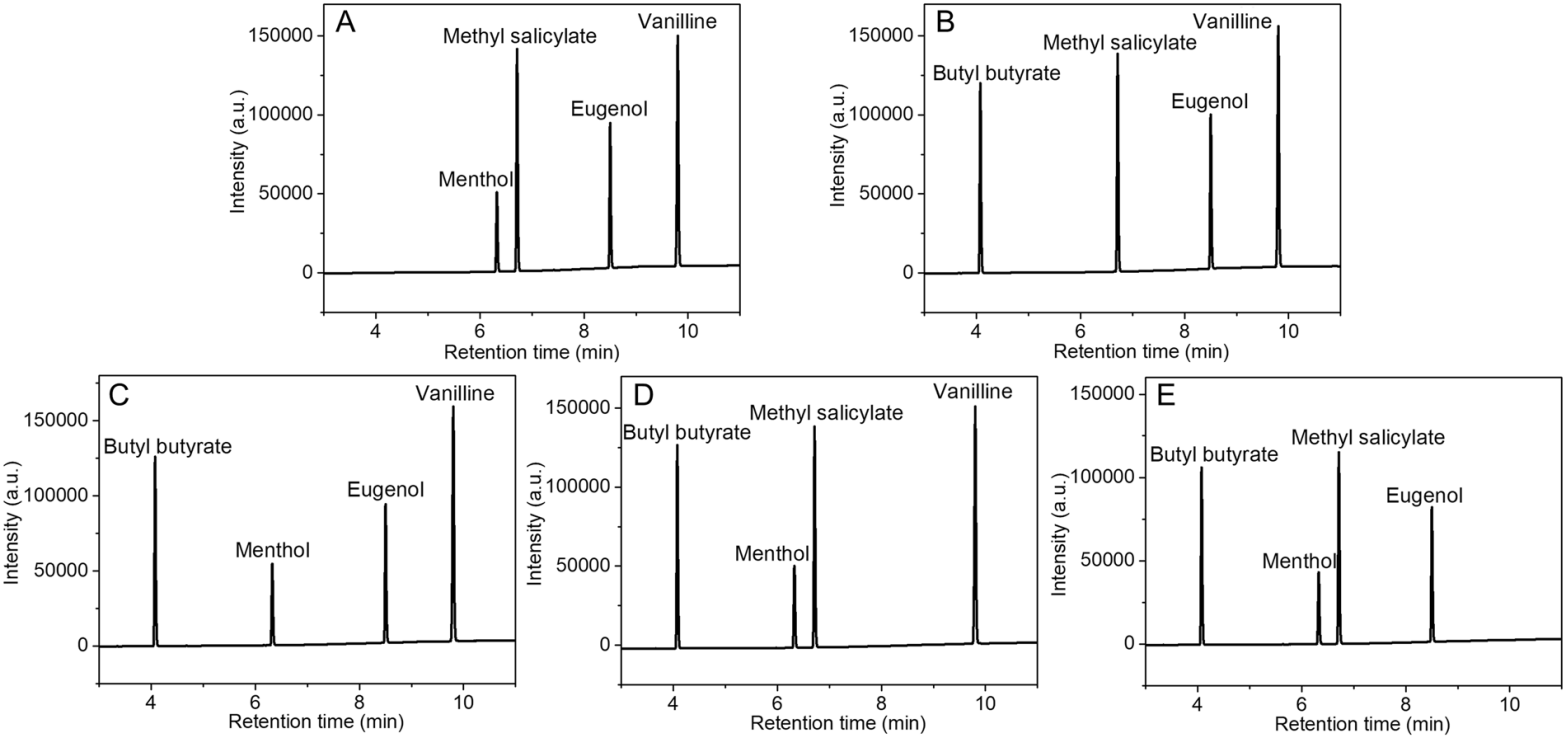
Gas chromatograms of four flavor agents without butyl butyrate (**A**), menthol (**B**), methyl salicylate (**C**), eugenol (**D**) and vanilline (**E**).

To verify the specificity of the analysis method and to exclude the influence of other substances on the determination. Five control solutions were used and each solution was prepared by mixing the standard solution of four of the five targets. Figure 2 illustrated that the five targets could be not interfered with each other in the chromatographic analysis. Therefore, the proposed method showed good selectivity for the five targets.

### Chromatogram of the representative sample

**Figure 3:**
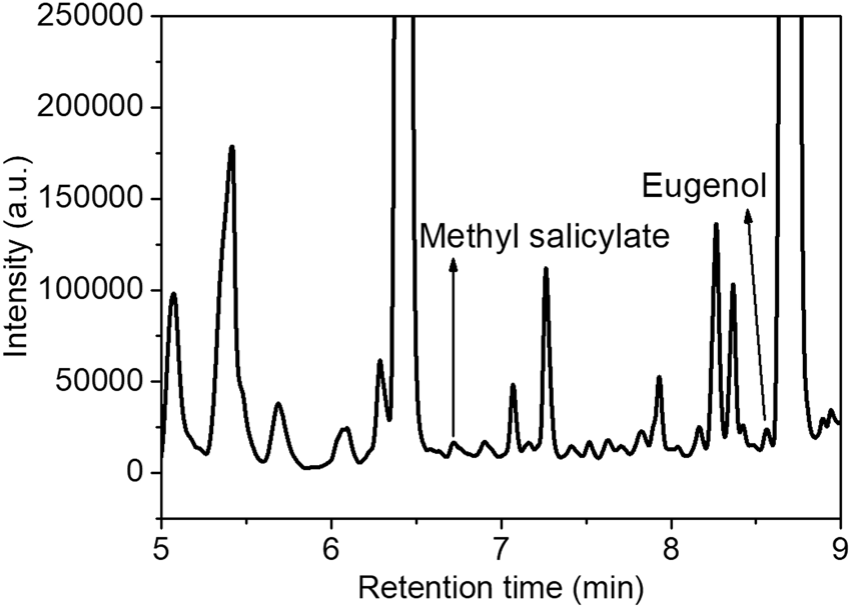
Gas chromatograms of strawberry-gum extracts.

To validate the applicability of the GC method for real sample assay, we tested the prepared flavor agents extracted from chewing gum. Actually, there are many different flavors of chewing gum on the market owing to their different spice ingredients. As an example, strawberry-gum was chosen as the model real sample. Figure 3 showed a representative chromatogram of the flavor agents extracted from strawberry-gum sample. Under chromatographic conditions listed in Table 1, two flavor agents, methyl salicylate and eugenol, were identified in the strawberry-gum samples and their retention times were 6.71 min and 8.50 min, respectively (Fig. 3). Through the coupled MS, the corresponding values of *m/z* were detected to be 152.07 and 164.10 for the above retention times, which were consistent with the theoretical molecular weight of methyl salicylate and eugenol. Although some unresolved peaks were apparent, which could presumably be other ingredients in chewing gum, methyl salicylate and eugenol contained in the samples could be easily separated and quantified, demonstrating that the described method had a good effect of qualitative and quantitative analysis for the real samples.

## Detection of Five Flavor Agents

The performance of this method for five flavor agents analysis was evaluated by determining different concentrations of each target flavor agent. The resulted data were summarized in Table 4. Calibration curves and regression equations of five targets were obtained by using a series concentrations of standard solutions. The regression equations for butyl butyrate, menthol, methyl salicylate, eugenol and vanilline were *A* = 7.81 × 10^6^*c*-5.8 × 10^4^, *A* = 9.96 × 10^6^*c*-7.3 × 10^4^, *A* = 5.71 × 10^6^*c*-7.8 × 10^4^, *A* = 8.90 × 10^6^*c*-4.1 × 10^4^ and *A* = 4.93 × 10^6^*c*-1.9 × 10^5^, respectively, where *c* represented concentration and A was peak area of the targets. For quantitative analysis, all the calibration curves showed good linear relationship with the concentrations ranged from 0.001 to 1.00 mg mL^−1^ for butyl butyrate, methyl salicylate and vanilline, from 0.001 to 0.40 mg mL^−1^ for menthol, and from 0.001 to 0.60 mg mL^−1^ for eugenol. The regression coefficients (R) for butyl butyrate, menthol, methyl salicylate, eugenol and vanilline were 0.9951, 0.9996, 0.9996, 0.9999 and 0.9974, respectively. Obviously, all of R values were more than 0.9900, indicating that the quantitative detection of the targets was accurate. The limits of detection (LODs) were 0.05 µg mL^−1^ (S/N = 3) for butyl butyrate, menthol, methyl salicylaet, and 0.09 µg mL^−1^ for eugenol and vanilline, which was similar with common GC-FID method (0.05 µg mL^−1^ menthol)^25^ but lower than capillary GC-FID method (0.2 µg mL^−1^ eugenol)^12^. The above results confirmed that the proposed method of ultrasound-microwave synergistic extraction coupled with GC were reliable and appropriate for the detection of flavoring agents in chewing gums.

**Table 4:** Calibration curves of five analytes in ethanol and performance parameters.

Analytes	Slope (95% confidence interval)	Intercept (95% confidence interval)	Regression coefficient (R)	Linear range (mg mL^−1^)	LODs (µg mL^−1^)
Butyl butyrate	(7.81 ± 0.023) × 10^6^	(−5.8 ± 0.20) × 10^4^	0.9951	0.001~1.00	0.05
Menthol	(9.96 ± 0.021) × 10^6^	(−7.3 ± 0.22) × 10^4^	0.9996	0.001~0.40	0.05
Methyl salicylate	(5.71 ± 0.028) × 10^6^	(−7.8 ± 0.22) × 10^4^	0.9996	0.001~1.00	0.05
Eugenol	(8.90 ± 0.020) × 10^6^	(−4.1 ± 0.36) × 10^4^	0.9999	0.001~0.60	0.09
Vanilline	(4.93 ± 0.021) × 10^6^	(−1.9 ± 0.20) × 10^5^	0.9974	0.001~1.00	0.09

### Performance evaluation in real samples assay

There generally is not information about favor compounds in the ingredients list of commercialized chewing gum. Moreover, these flavoring agents cannot be accurately determined by traditional method. Therefore, we performed standard recovery method and parallel determination experiment to determine the precision and accuracy of the method. Taking a gum without any volatile flavor compounds as a model, we researched the feasibility of the proposed method and the results were summarized in Table 5. The measured relative standard deviation (RSD) ranged within 3.6−5.2% and the recoveries were calculated to be within 95.7−103.4%, indicating the method had good accuracy and repeatability. To further confirm the reliability of the proposed method, standard recovery method on real samples assay in strawberry-gum (Table 6). The measured relative standard deviations ranged within 3.1−5.6% and the recoveries were calculated to be within 90.7−102.4%, confirming that this method could be qualified for extraction and determination of the flavoring agents in chewing gum.

**Table 5:** Detection of five flavor agents in chewing gum without any volatile flavor compounds.

Analytes	Detected (μg/g)	Added (μg/g)	Found (μg/g)	Recovery (%)	RSD (%, *n* = 5)
Butyl butyrate	0	121.0	115.8	95.7	4.5
Menthol	0	85.88	83.48	97.2	3.7
Methyl salicylate	0	115.8	117.4	101.4	3.6
Eugenol	0	89.47	87.08	97.3	4.9
Vanilline	0	84.28	87.08	103.3	5.2

**Table 6:** Real samples assay in strawberry-gum.

Analytes	Detected (μg/g)	Added (μg/g)	Found (μg/g)	Recovery (%)	RSD (%, *n* = 5)
Butyl butyrate	0	120.9	116.1	96.0	3.8
Menthol	0	85.76	81.77	95.3	3.1
Methyl salicylate	24.73	115.7	143.2	102.4	3.9
Eugenol	7.180	89.35	88.95	91.5	4.2
Vanilline	0	84.16	76.18	90.5	5.6

## Conclusion

The reliable detection of the food additives is of significant importance to ensure food safety and concordance with regulations. In this work, a new method for the simultaneous determination of five flavoring agent contents in chewing gum was established by the ultrasound-microwave synergistic extraction combined with GC technology. The flavor agents were effectively extracted from the chewing gum samples through ultrasound-microwave synergistic extraction. Furthermore, five flavoring agents were simultaneous identified and determined by GC technology. The statistical results demonstrated that the proposed method was efficient and practical for the determination of the flavoring agents in chewing gum and other foods. Therefore, the proposed ultrasound-microwave synergistic extraction coupled with GC technology would hold great promise for application in the food safety assay.

## Materials and Methods

### Chemicals and materials

Butyl butyrate, menthol, vanilline, methyl salicylate and eugenol standards were purchased from Sigma-Aldrich (Beijing, China). Ethanol of liquid-chromatography grade was purchased from Merck (Shanghai, China). Chewing gum were obtained from local markets in Qingdao city of Shandong Province. According to Fierens *et al*.^26^, sample contamination can occur in every stage of the analytical procedure because it is ubiquitous in laboratory environment. Therefore, all the glassware was thoroughly washed and rinsed with deionized water and acetone prior to use. Standard stock solutions of butyl butyrate, menthol, vanilline, methyl salicylate and eugenol were prepared in ethanol. Standard working solutions were prepared by the stock solution with serial dilution. All solutions were stored in amber vials at 4 °C for later use.

### Apparatus

The gas chromatograph system GC-2010 plus (Shimadzu, Japan) equipped with a FID and an AOC-20i automatic sample injector. The Rtx-1701 capillary column (length = 30 m, inner diameter = 0.25 mm with thickness of stationary phase = 0.25 μm, Restek, USA) was used for separation. LabSolutions workstation (Shimadzu, Japan) was used to control the GC system and manipulate the chromatograms. The CW-2000 ultrasound-microwave synergistic extraction generator from the Shanghai XTrust Analytical instrument technology Co., Ltd. (Shanghai, China) was used to extract the objective compounds in chewing gum. The high-speed tabletop centrifuge (Anting Corp., Shanghai, China) and high speed universal pulverizer from the Taisite Instrument Co., LTD. (Tianjin, China) were used during sample preparation. The rotary vacuum evaporator from the Yarong biochemical instrument factory (Shanghai, China) was used to evaporate the supernatants.

### Conditions for chromatographic separation

Gas chromatographic separation was performed with FID and split/splitless injection mode. The optimized chromatographic conditions were followed in Table 1.

### Sample preparation

Sample preparation is one of the most important procedures to remove the food interferences and enrich the analytes, especially in the case of complex component foods. In general, homogenized food is used for the extraction process. Typically, the chewing gum samples were firstly frozen at −20 °C for 2 h to facilitate crushing and crushed into small particles with a universal pulverizer. Then the weighed powdered chewing gum sample (2.5 g) were added into a microwave flask equipped containing liquid chromatography grade ethanol (50 mL) with a reflux condenser and the temperature sensed by infrared sensor was set to be 60 °C. The mixture was irradiated with microwave power at 400 W for 30 min ultrasound-microwave synergistic extraction. The status of the extraction was observed by a video liquid crystal monitors. After completion of synergistic extraction, the mixture was transferred to the centrifuge tubes and centrifugated for 10 min at 10000 rpm. Then, the liquid supernatants of the synergistic extracted mixture were collected. All supernatants were merged together and evaporated to dryness by a rotary vacuum evaporator. The final solid residue was dissolved in 2 mL liquid chromatography grade ethanol. The detection of trace targets in the sample can be guaranteed by enriching and concentrating 25 times for these targets. Finally 2 mL solution was filtered by polytetrafluoroethylene syringe filters with a pore size of 0.45 μm before gas chromatographic analysis.

### Real samples assay

Taking gums without any volatile flavor compounds as a model, we took standard recovery method to verify the accuracy of the method. The five flavoring agents (303 µg of butyl butyrate, 215 µg of menthol, 290 µg of methyl salicylate, 224 µg of eugenol, 211 µg of vanilline) were respectively added into the above gums (about 2.5 g). The samples were prepared according to the above-mentioned method of sample preparation, and the determination was carried out according to gas chromatograph conditions of Table 3. The recoveries and RSD were calculated in accordance with the results of gas chromatographic method. Subsequently, standard recovery method was also performed on real samples assay in strawberry-gum by the above-mentioned process.

## References

[1] Wang ZY (2016). Determination of vanillin and ethyl-vanillin in milk powder by headspace solid-phase microextraction coupled with gas chromatography-mass spectrometry. Food Anal. Method..

[2] Corbi E, Pérès C, David N (2014). Quantification of furocoumarins in hydroalcoholic fragrances by a liquid chromatography-high resolution/accurate mass method. Flavour Frag. J..

[3] Peng QR (2013). Determination of volatile phenols in Chinese liquors by high-performance liquid chromatography associated with beta-cyclodextrin and a protective barrier layer. Flavour Frag. J..

[4] Sharma UK, Sharma N, Sinha AK, Kumar N, Gupta AP (2009). Ultrafast UPLC-ESI-MS and HPLC with monolithic column for determination of principal flavor compounds in vanilla pods. J. Sep. Sci..

[5] Xiao R (2017). Analysis of flavors and fragrances by HPLC with Fe3O4@GO magnetic nanocomposite as the adsorbent. Talanta.

[6] Anne O, Jüri K (2001). Determination of peppermint and orange aroma compounds in food and beverages. P. Est Acad. Sci..

[7] Botrel BMC (2017). Residual determination of anesthetic menthol in fishes by SDME/GC–MS. Food Chem..

[8] Li JC, Zhang J, Liu Y (2015). Optimization of solid-phase-extraction cleanup and validation of quantitative determination of eugenol in fish samples by gas chromatography–tandem mass spectrometry. Anal. Bioanal. Chem..

[9] Li JC, Liu H, Wang CY, Wu LD, Liu D (2016). Determination of eugenol in fish and shrimp muscle tissue by stable isotope dilution assay and solid-phase extraction coupled gas chromatography–triple quadrupole mass spectrometry. Anal. Bioanal. Chem..

[10] Liang X (2018). Vortex-assisted liquid-liquid micro-extraction followed by head space solid phase micro-extraction for the determination of eugenol in fish using GC-MS. Food Anal. Method..

[11] Goodner KL, Jella P, Rouseff RL (2000). Determination of vanillin in orange, grapefruit, tangerine, lemon, and lime juices using GC-olfactometry and GC-MS/MS. J. Agr. Food Chem..

[12] Yu BS, Lai SG, Tan QL (2006). Simultaneous determination of cinnamaldehyde, eugenol and paeonol in traditional chinese medicinal preparations by capillary GC-FID. Chem. Pharm. Bull..

[13] Valdez JS, Martin DK, Mayersohn M (1999). Sensitive and selective gas chromatographic methods for the quantitation of camphor, menthol and methyl salicylate from hum an plasma. J. Chromatogr. B.

[14] De Jager LS, Perfetti GA, Diachenko GW (2008). Comparison of headspace-SPME-GC–MS and LC–MS for the detection and quantification of coumarin, vanillin, and ethyl vanillin in vanilla extract products. Food Chem..

[15] Huang ZH (2015). Simultaneous determination of salicylic acid, jasmonic acid, methyl salicylate, and methyl jasmonate from ulmus pumila leaves by GC-MS. Int. J. Anal. Chem..

[16] Lin RM, Tian J, Huang G, Li TC, Li FM (2002). Analysis of menthol in three traditional Chinese medicinal herbs and their compound formulation by GC-MS. Biomed. Chromatogr..

[17] Badwaik LS, Borah PK, Deka SC (2015). Optimization of microwave assisted extraction of antioxidant extract from garcinia pedunculata robx. Sep. Sci. Technol..

[18] Bayramoglu B, Sahin S, Sumnu G (2009). Extraction of essential oil from laurel leaves by using microwaves. Sep. Sci. Technol..

[19] Nayak B (2015). Comparison of microwave, ultrasound and accelerated-assisted solvent extraction for recovery of polyphenols from Citrus sinensis peels. Food Chem..

[20] Mane S, Bremner DH, Tziboula-Clarke A, Lemos MA (2015). Effect of ultrasound on the extraction of total anthocyanins from Purple Majesty potato. Ultrason. Sonochem..

[21] Morsy NFS (2015). A short extraction time of high quality hydrodistilled cardamom (Elettaria cardamomum L. Maton) essential oil using ultrasound as a pretreatment. Ind. Crop. Prod..

[22] Liu ZZ, Gu HY, Yang L (2015). An approach of ionic liquids/lithium salts based microwave irradiation pretreatment followed by ultrasound-microwave synergistic extraction for two coumarins preparation from Cortex fraxini. J. Chromatogr. A.

[23] Yang YQ (2016). Rapid determination of the volatile components in tobacco by ultrasound-microwave synergistic extraction coupled to headspace solid-phase microextraction with gas chromatography-mass spectrometry. J. Sep. Sci..

[24] Wang YQ, Huo XW (2018). Multiobjective optimization design and performance prediction of centrifugal pump based on orthogonal test. Adv. Mater Sci. Eng..

[25] Nozal MJ, Bernal JL, Jimenez JJ, Gonzalez MJ, Higes M (2002). Extraction of thymol, eucalyptol, menthol, and camphor residues from honey and beeswax - Determination by gas chromatography with flame ionization detection. J. Chromatogr. A.

[26] Fierens T (2012). Analysis of phthalates in food products and packaging materials sold on the Belgian market. Food Chem. Toxicol..

